# Low-cost electron detector for scanning electron microscope

**DOI:** 10.1016/j.ohx.2023.e00413

**Published:** 2023-03-10

**Authors:** Evgenii Vlasov, Nikita Denisov, Johan Verbeeck

**Affiliations:** Electron Microscopy for Materials Science, University of Antwerp, Groenenborgerlaan 171, 2020 Antwerp, Belgium

**Keywords:** Electron detection, Scanning electron microscopy, Open-source

## Abstract

Electron microscopy is an indispensable tool for the characterization of (nano) materials. Electron microscopes are typically very expensive and their internal operation is often shielded from the user. This situation can provide fast and high quality results for researchers focusing on e.g. materials science if they have access to the relevant instruments. For researchers focusing on technique development, wishing to test novel setups, however, the high entry price can lead to risk aversion and deter researchers from innovating electron microscopy technology further. The closed attitude of commercial entities about how exactly the different parts of electron microscopes work, makes it even harder for newcomers in this field. Here we propose an affordable, easy-to-build electron detector for use in a scanning electron microscope (SEM). The aim of this project is to shed light on the functioning of such detectors as well as show that even a very modest design can lead to acceptable performance while providing high flexibility for experimentation and customization.

## Graphical abstract

**Figure:**
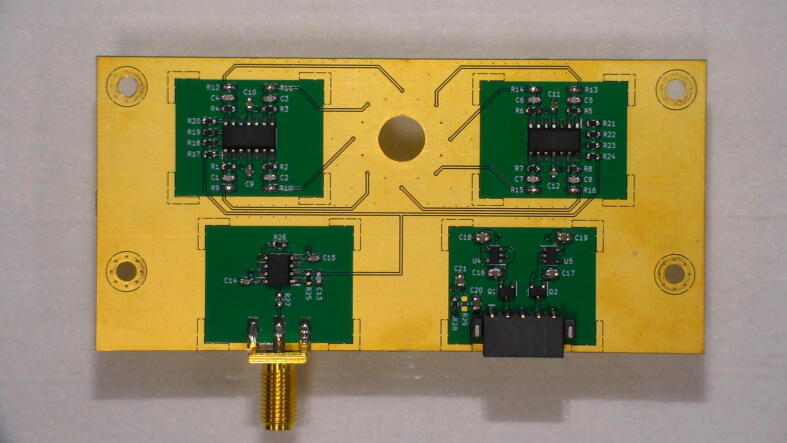

**Specifications table**

**Table:** 

**Hardware name**	ADF/BSE electron detector for scanning electron microscopy
**Subject area**	• Engineering and material science
**Hardware type**	• Imaging tools • Measuring physical properties and in-lab sensors • Mechanical engineering and materials science
**Closest commercial analog**	• BSE detector by point electronic GmbH • REBEKA and KARMEN detectors by CRYTUR Ltd • BSE and HAADF SEM/STEM detector by Deben UK Ltd • Detectors by EM vendors (ThermoFisher, Jeol, Hitachi, Tescan, etc.)
**Open source license**	CC BY-SA 4.0 (Creative Commons Attribution-ShareAlike 4.0 International)
**Cost of hardware**	Approx. 100 EUR
**Source file repository**	https://doi.org/10.5281/zenodo.7474569

## 1 Hardware in context

Electron microscopy (EM) is a powerful analytical technique that allows to study materials at the micro- and nanoscale [1], [2], [3]. It uses a high-energy beam of electrons to produce images of samples with a much higher resolution than is possible with a traditional optical microscope. Signals generated during electron-matter interaction convey information about the specimen structure, morphology, and composition. The capability to provide high-resolution images and information on materials’ physical and chemical properties makes EM an invaluable tool for a wide range of scientific and industrial applications.

EM is an actively developing field of modern science [4]. The recent developments include a number of new imaging techniques [5], [6], the use of advanced detectors [7], and the development of new machine learning algorithms to analyze and interpret electron microscopy data [8]. However, EMs and their complementary equipment including electron detectors are extremely expensive and their internal operation is often entirely hidden from the researchers. This poses barriers to innovation and also leads to an education gap where researchers have no access to how their instruments work and how this could affect their observations.

The use of open-source hardware designs can drastically reduce the cost of scientific research and experimentation. This is especially beneficial for small research groups that may not have the budget to purchase proprietary hardware. Open hardware encourages collaboration and sharing within the scientific community. Researchers can build upon existing designs and contribute their own modifications, leading to faster progress and innovation. The designs can be customized and modified according to specific needs or requirements. This can be especially useful for creating specialized scientific instruments or adapting hardware for specific experiments.

Here we propose a design of a low-cost (< 100 EUR) solid-state electron detector that can be used for capturing signals in EM namely backscattered (BSE) and transmitted electrons (TE) in annular dark-field (ADF) mode. The design was optimized for noise rejection and detection of relatively low signals.

## 2 Hardware description

**Fig. 1:**
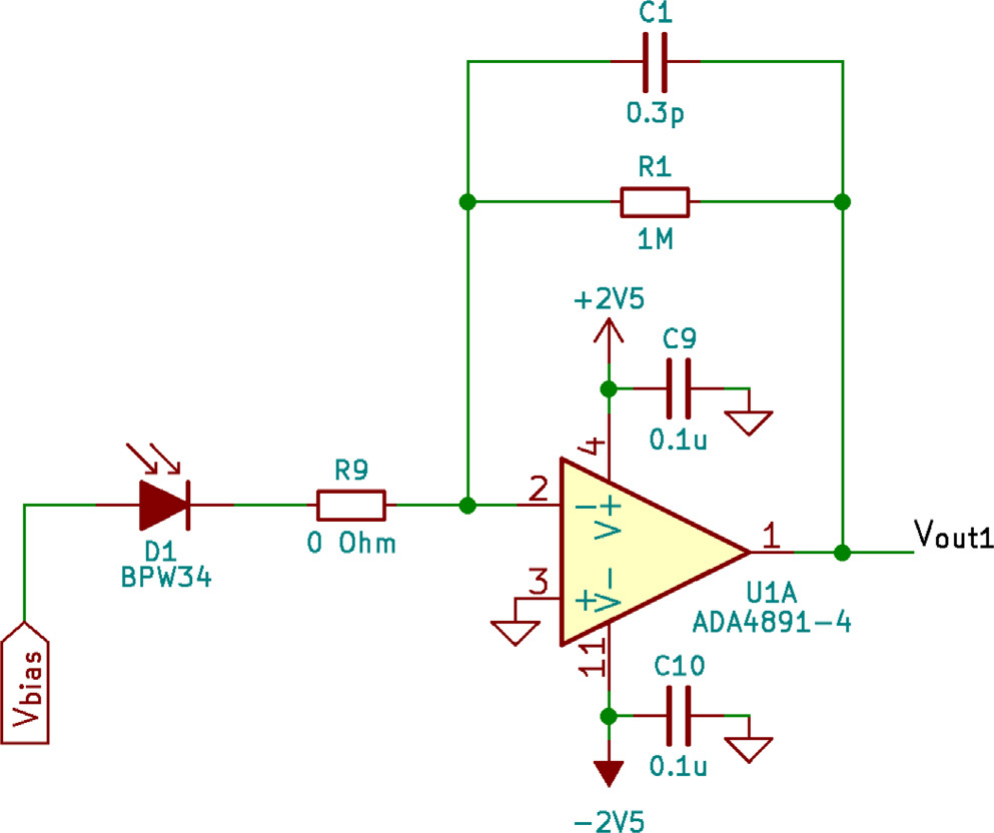
Transimpedance amplifier (TIA). TIA network comprises 8 of such circuits.

**Fig. 2:**
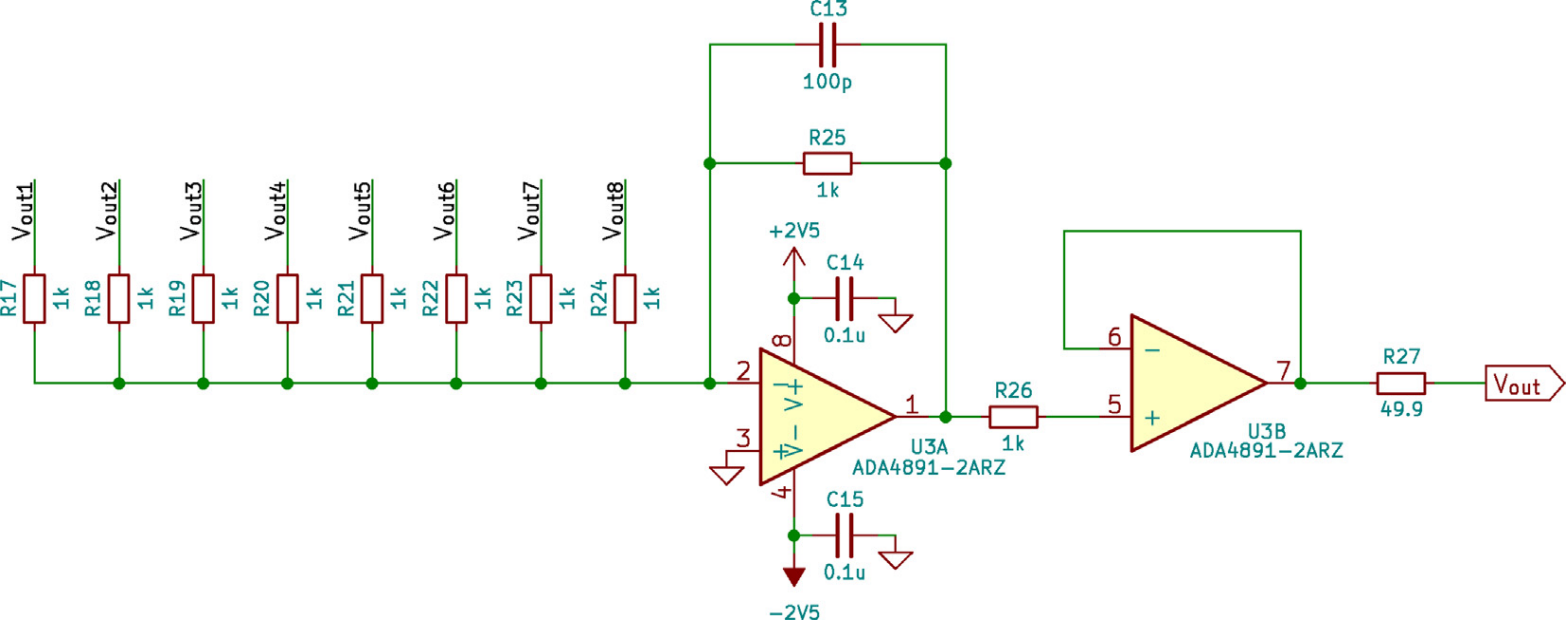
Summing amplifier stage.

The four-layer printed circuit board (PCB) was designed using KiCad 6.0.0. The design files can be found in the section below (see. the Design files section). The detector comprises several elements: (1) photodiode sensing array; (2) pre-amplifier stage (a series of transimpedance amplifiers (TIA)); (3) a summing amplifier; and (4) power supply (Fig. 1, Fig. 2). The detector array consists of eight Si photodiodes (BPW34, Vishay) azimuthally arranged around the electron beam. In the current implementation the eight photodiode signals are summed to emulate a larger and oddly shaped area detector without the expense that would come with a custom semiconductor design. An added benefit of this choice is that with only minor modifications also segmented readout becomes possible as discussed at the end of this manuscript. The photodiodes were modified in order to expose a Si chip making it sensitive to a direct electron radiation. Photodiodes convert high-energy electrons reaching their depletion region into electron-hole pairs ($∼10^{3}$ electron-hole pairs generated per 1 detected electron). We have chosen BPW34 Si photodiodes as a detector since they are affordable (0.68 EUR per part), have a large sensitive area ($3\times 3{\mathrm{mm}}^{2}$), and relatively low capacitance (40 pF at 3 V reverse bias voltage). This type of PIN photodiode was previously used in a number of open-source radiation detectors. (e.g. [9]).

Each photodiode is connected to its individual TIA with a gain of $10^{6}$ V/A which is defined by the resistance of a feedback resistor (e.g. $R_{1}$). The gain settings were chosen accordingly to amplify the signal to a reasonable level that can be handled by e.g. the scan engine of the microscope. The TIA operates with an ADA4891-4 quad op-amp (Analog Devices) which can offer both low bias input current and high speed required for the design. In a TIA design, a feedback capacitor (e.g. $C_{1}$) is crucial to limit the transfer function of the feedback loop to the region where phase stability is guaranteed [10]. In our design $C_{1}$ value was chosen as 0.3 pF to create a cutoff frequency of $f=\frac{1}{2\pi R_{1}C_{1}}\approx$ 530 kHz, and gain of $10^{6}$.

The outputs of the TIA’s then are connected to a summing amplifier (Fig. 2) followed by a voltage follower in order to match the impedance of the output of the detector with a coaxial cable.

**Fig. 3:**
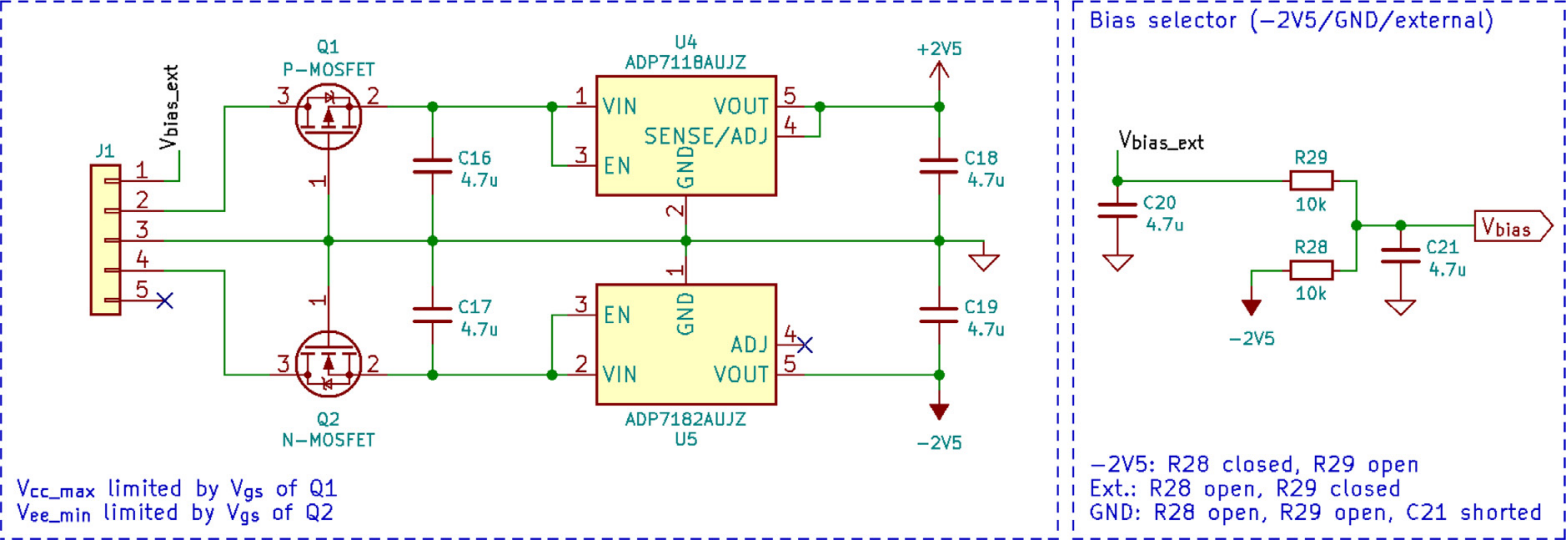
Schematic of power supply stage and bias selector.

A power supply stage (Fig. 3) of the detector offers reverse polarity protection built on P- and N-channel MOSFETs $Q_{1}$ and $Q_{2}$. It consists of positive and negative low-dropout voltage regulators $U_{4}$ and $U_{5}$ that convert the input voltage to ±2.5 V needed for the operation of the operational amplifiers. The design provides low noise, and achieves excellent line and load transient response. To reverse bias photodiode sensors, we implemented a circuit allowing switching between -2.5 V, an external bias voltage (BPW34 can be biased to −60 V max), and GND.

Our detector design provides the following features:

• Low cost
• Transimpedance gain for each sensor: $10^{6}$ V/A
• High bandwidth (approx. 550 kHz)

## 3 Design files summary

The design files consist of the documents needed to order and assemble the board. All Kicad files are created with V6.0.0. It includes the following files:

**Table:** 

**Design filename**	**File type**	**Open source license**	**Location of the file**
BSE detector.kicad_pro	KiCAD project	CC BY-SA 4.0	https://doi.org/10.5281/zenodo.7474569
BSE detector.kicad_sch	KiCAD schematic	CC BY-SA 4.0	https://doi.org/10.5281/zenodo.7474569
TIA network.kicad_sch	KiCAD schematic	CC BY-SA 4.0	https://doi.org/10.5281/zenodo.7474569
Power supply.kicad_sch	KiCAD schematic	CC BY-SA 4.0	https://doi.org/10.5281/zenodo.7474569
BSE detector.kicad_brd	KiCAD pcb layout	CC BY-SA 4.0	https://doi.org/10.5281/zenodo.7474569
Gerber.zip	Fabrication file	CC BY-SA 4.0	https://doi.org/10.5281/zenodo.7474569
BOM.xlsx	Excel file	CC BY-SA 4.0	https://doi.org/10.5281/zenodo.7474569

## 4 Bill of materials summary

The complete bill of materials can be found on the Zenodo repository. The excel document includes all required components. We have chosen feedback resistors with a tolerance of 0.1% to provide the highest accuracy of TIA gain.

## 5 Build instructions

**Fig. 4:**
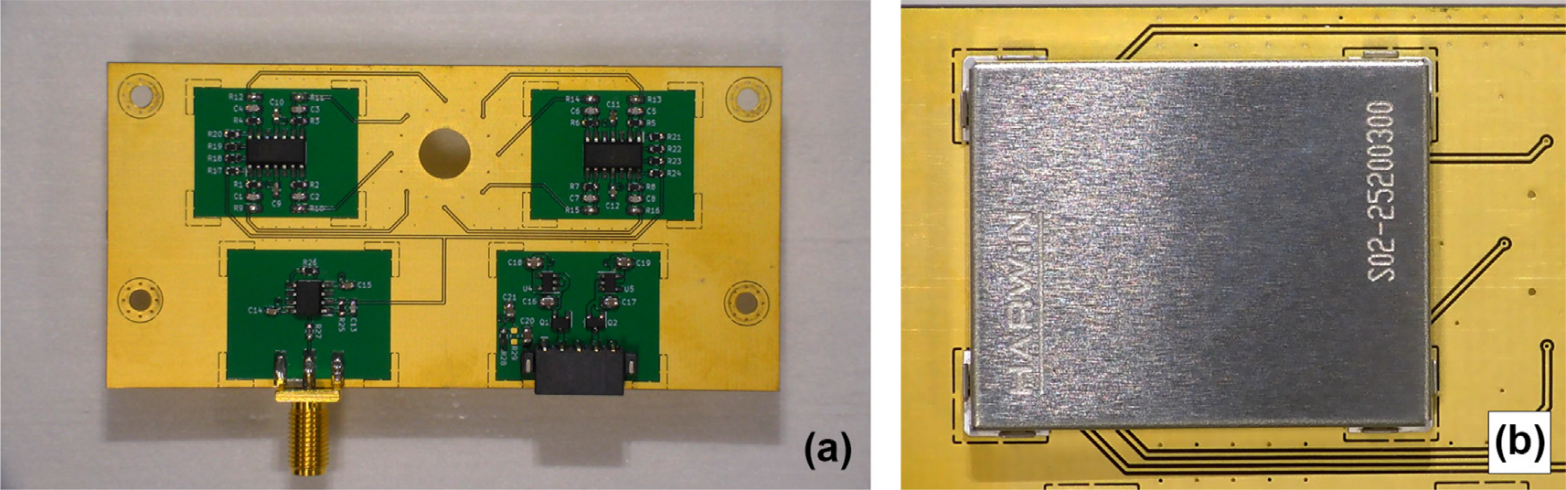
(a) Assembled PCB. For visibility purposes, the radio frequency shield has not been soldered to the board in this picture, (b) attached the radio frequency shield.
• Set up the bias selector by selecting a combination of $R_{28},R_{29}$ and $C_{21}$ according to Fig. 3.
• Thoroughly clean assembled PCB with isopropanol to avoid any undesirable contamination.
• Attach thin wires connecting the anodes of the photodiodes to the corresponding pads of the PCB. Use either a wire bonding machine or manual bonding with silver paint. Fig. 5 shows an SEM image of a wire-bonded photodiode.

• Remove the photodiode chip from its plastic package using dichloromethane. The procedure may take more than 12 h before the package becomes fragile and can be easily peeled off to expose the Si chip. Be aware of safety measures working with dichloromethane. Note that the procedure destroys the wire connection of the anode to the corresponding lead that needs to be restored at a later stage. Cut off the remaining photodiode lead (cathode).
• Populate the PCB using a soldering iron, hot air gun, or reflow oven for surface-mount device (SMD) components. We recommend using low-temperature soldering (<260°C) for the photodiodes to avoid damaging them. The assembled PCB is shown on Fig. 4.

**Fig. 5:**
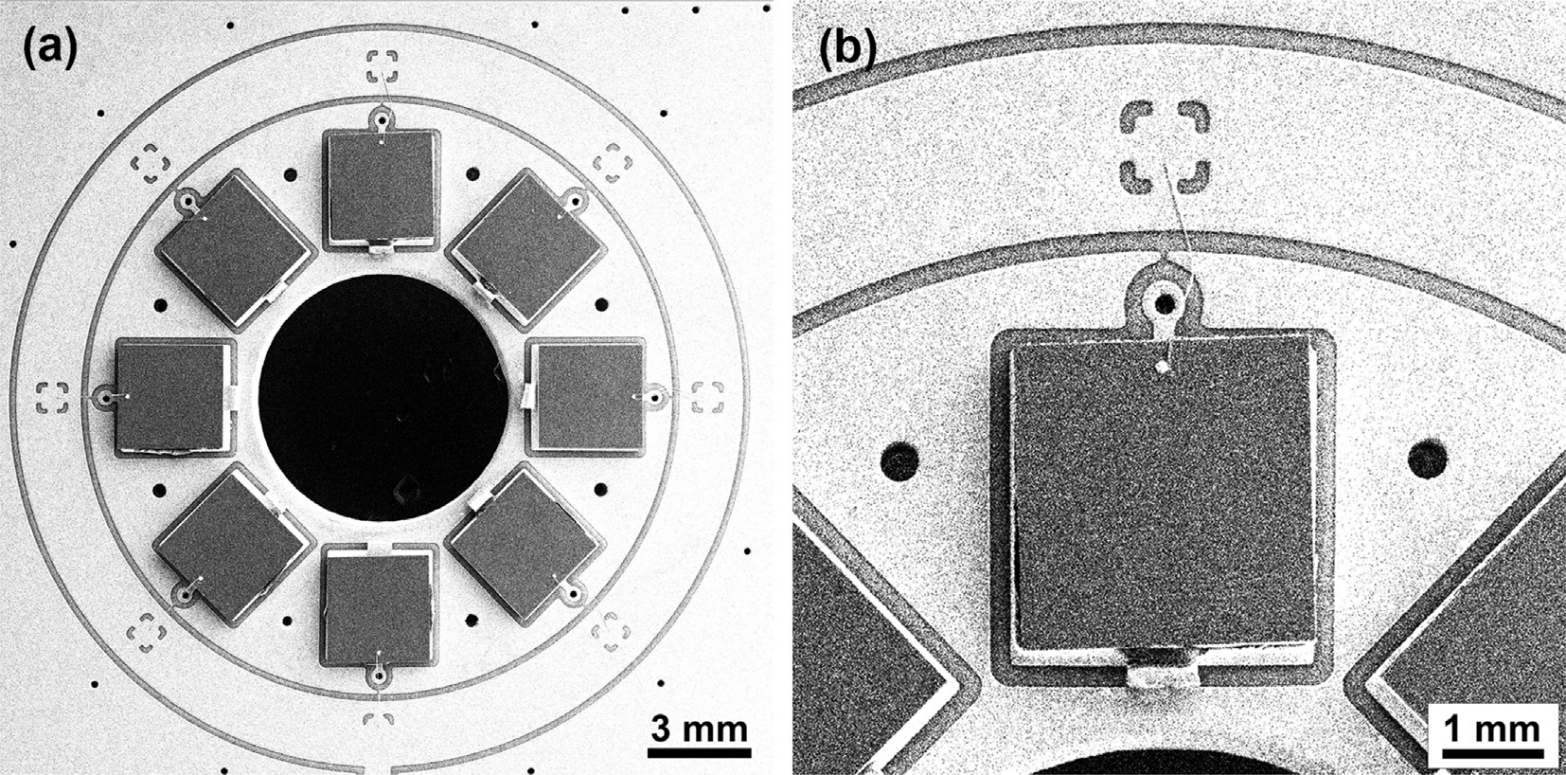
(a) overview SEM image of sensitive detector area; (b) SEM image of individual photodiode.
• If required, the radio frequency shields can be installed to reduce susceptibility to external fields (Fig. 4 b).
• Install standoffs at the four corners of the PCB if needed and figure out a way to mount the detector in the SEM chamber.

## 6 Operation instructions

• Find a way to create electrical feedthrough compatible with your microscope for at least 3 wires for powering the device (with proper overcurrent protection) and 1 coaxial cable for the signal. This part is not included in the current design as the mechanics are entirely microscope-specific and proper care needs to be taken in terms of X-ray safety. We recommend working with the existing feedthrough possibilities of your SEM or going through proper X-ray shielding tests with a certified body.
• Connect a symmetrical power supply (not included in design) using $J_{1}$ pluggable terminal block connector. Since the detector is designed to be operated in a vacuum chamber of a scanning electron microscope, we recommend using ±3.3 V or ±3 V power input to reduce the power dissipation in the voltage regulators as much as possible.
• Connect the $V_{\mathrm{out}}$ SMA connector ($J_{3}$) to scan engine via coax cable and adjust gain and offset settings of the latter (if applicable). In our experiments, we use an Attollight OUDS II scan engine.
• Turn off the IR LED inside the SEM chamber (if applicable) since it can cause saturation of the detector.
• Use board spacers or standoffs to avoid any unwanted short-circuit with a potentially conductive surface.

## 7 Validation and characterization

We have tested the capabilities of the detector for working in SEM conditions. First, an evaluation of the detector’s working range was performed. We have determined, that while illuminating the detector with a direct incident electron beam (without a sample in between the pole piece and detector) the signal on the detector appears at primary electron beam energies above 3 keV and it still can be detected up to 30 keV. Most modern SEMs can produce electron beams with an energy of up to 30 keV, thus the detector can be used in a large variety of instruments for the detection of high-energy electrons. We assume that the detector can be used even with substantially higher beam energies (e.g. in transmission EM conditions) but this has not been tested in this paper. However, others have tested the behaviour of these photodiodes (with plastic package intact) for a range of different particles beams with energies up to 1 MeV showing much wider operation range might be possible [11]. The lowest detected energy value of 3 keV can be explained by the presence of a dead layer inherent in solid-state radiation detectors. This makes the detector insensitive to slow electrons that cannot penetrate the dead layer of the photodiode. Therefore, the detector is insensitive to secondary electrons (typical energies below 50 eV). Beam current tests resulted in an operational range from 1 pA to 50 nA at 30 keV when the sensor is directly exposed to the primary electron beam.

Detector frequency bandwidth was estimated by processing intensity profiles for images of sharp-edge object taken with different scan speeds (different pixel dwell times). From this intensity profile, we extracted the “vacuum – object” transition area (step function) and determined the maximum rise-fall time of 623 ns which amounts to approx. 562 kHz bandwidth.

**Fig. 6:**
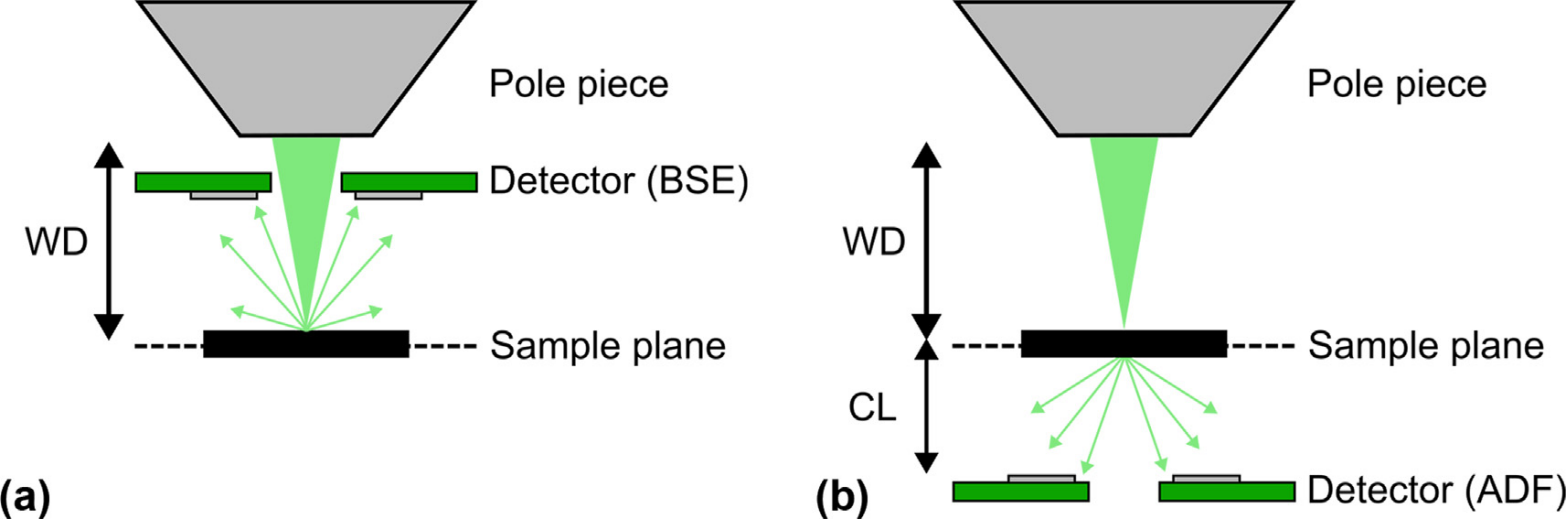
Schematic showing the geometry of the experiments during characterization of the detector: (a) BSE mode, (b) ADF mode. WD denotes a working distance, CL – camera length.

**Fig. 7:**
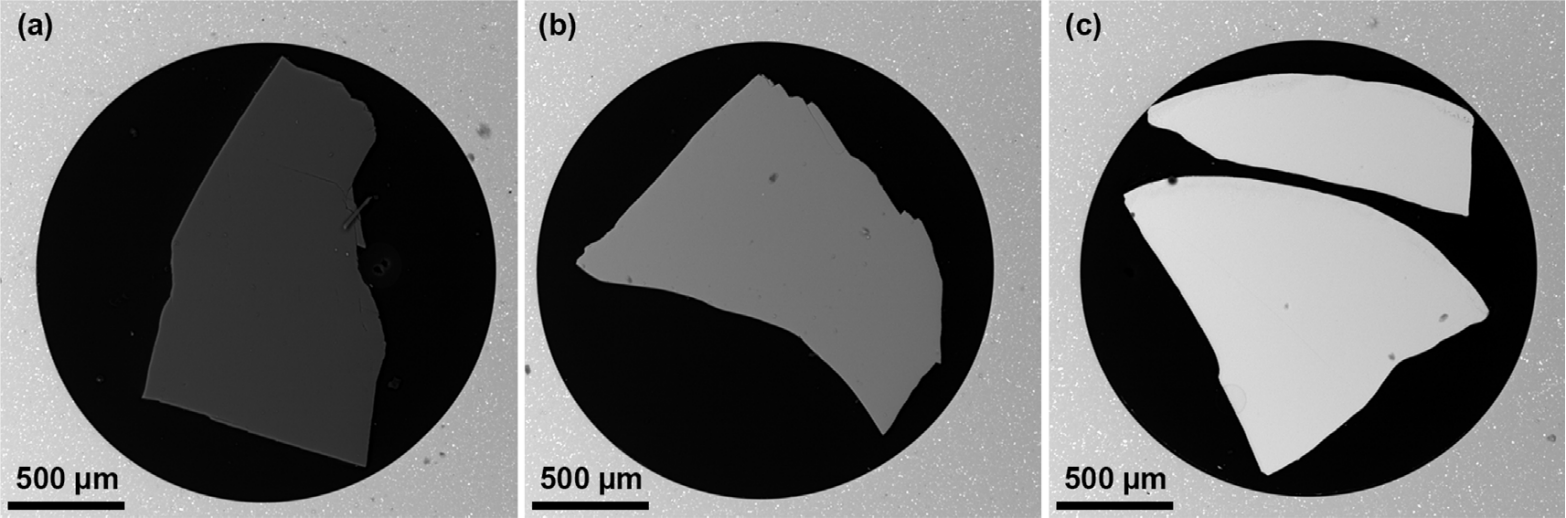
SEM images obtained in BSE mode of the detector for different materials: (a) orthoclase (KAlSi_3_O_8_), (b) pyrite (FeS_2_), (c) selenium.

We have tested the performance of the detector in BSE mode. The detector was mounted above the sample at a distance of 20 mm from the objective lens to collect the BSE signal (see Fig. 6). We utilized a standard sample that offers a variety of different elements on one wafer (Standard 45 by Micro-Analysis Consultants Ltd). Fig. 7 shows images of orthoclase (${\mathrm{KAlSi}}_{3}O_{8}$), pyrite (${\mathrm{FeS}}_{2}$) and selenium taken in BSE mode. It can be seen that with an increase in the element’s atomic number, the intensity of the image increases. Such dependence signifies that the detector indeed works as a BSE detector under these conditions.

**Fig. 8:**
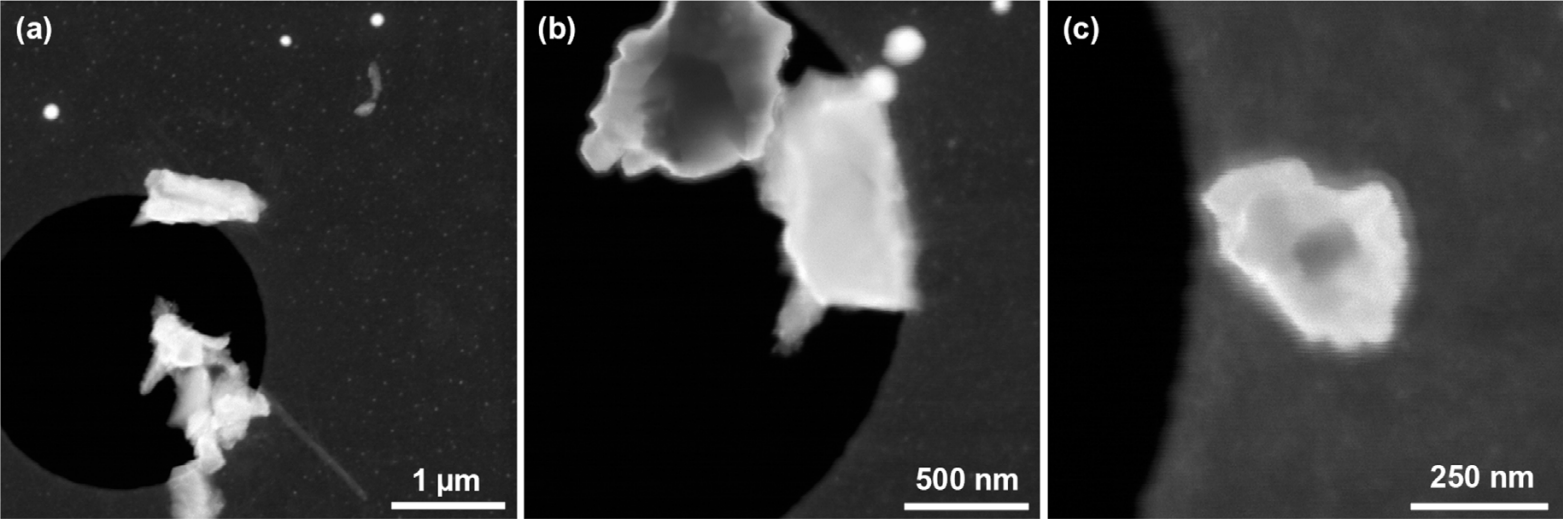
SEM images of CeO_2_ obtained with the detector in ADF mode at different magnifications: (a) 60 kx, (b) 135 kx, (c) 300 kx. The dark contrast within the particles can be explained by the absorption of the electrons due to the large thickness.

SEM in transmission mode (TSEM) is becoming a valuable technique that bridges the gap between SEM and transmission electron microscopy combining the versatility of SEM with advantageous imaging using transmitted electrons [12]. Therefore, the setup was re-configured as an ADF detector by placing the device below the sample to evaluate its capability for TE mode imaging. Images of CeO_2_ powder were acquired to demonstrate the mass-thickness behavior of the contrast obtained in ADF imaging mode (Fig. 8).

**Fig. 9:**
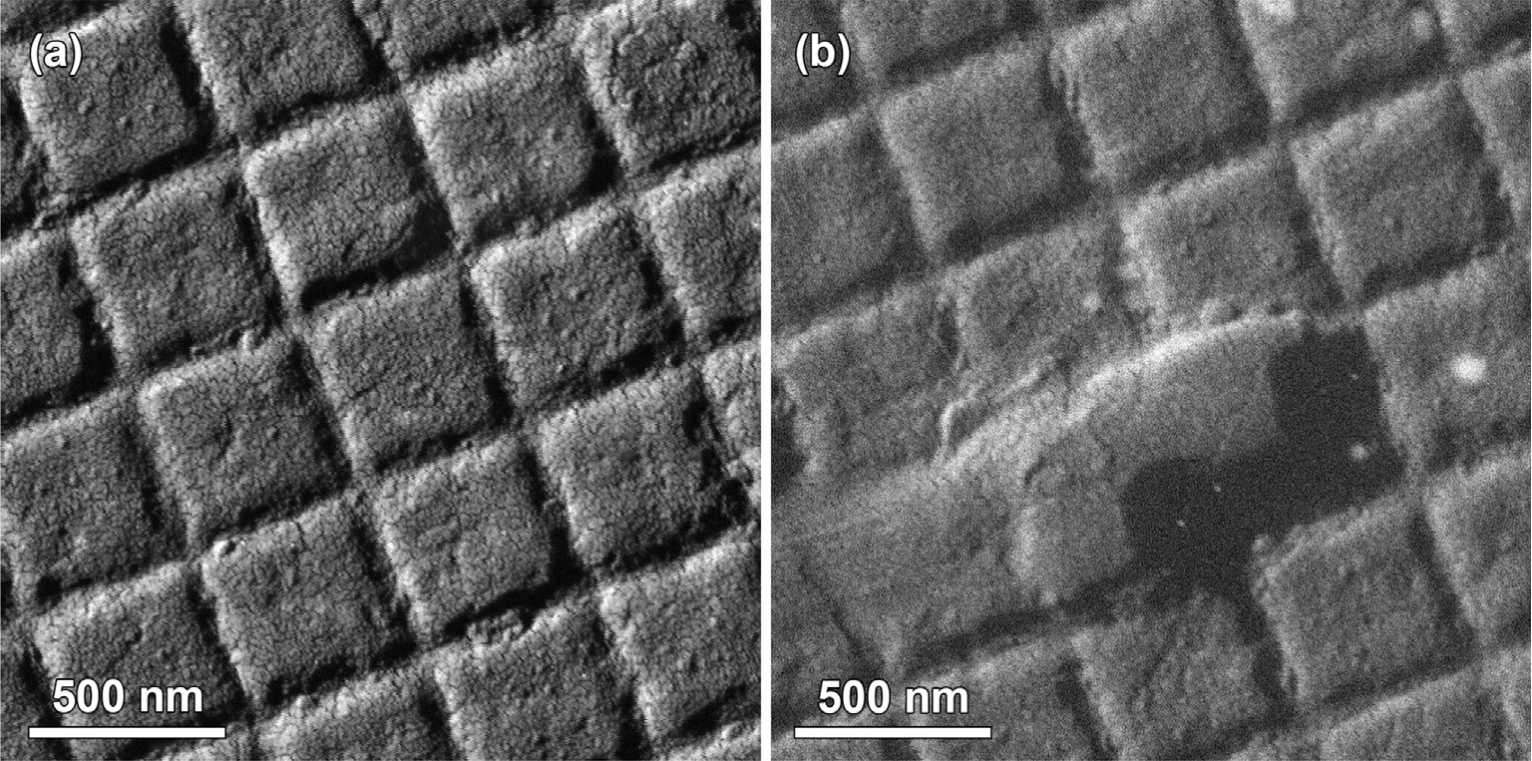
SEM images of Au cross-grating obtained with the detector in ADF mode in different vacuum modes: (a) High-Vac (4.6 mPa), (b) Low-Vac (700 Pa).

It should be noted that the main imaging mode in SEM uses secondary electron imaging to retrieve the information about the surface of the sample. However conventional Everhart–Thornley detectors (ETD) are not capable to work at low-vacuum pressures (as opposed to high-vacuum range) due to the risk of arcing. We have demonstrated that our detector design can overcome this limitation. In Fig. 9 we present SEM ADF images of a gold cross-grating taken in high-vacuum (4.6 mPa) and low-vacuum (700 Pa) modes. As one can see both pictures show distinguishable features of the sample which confirms the low-vac operation capabilities of the detector. The presence of gas molecules along the path of the electron beam creates more electron scattering and results in a less sharp image.

The parameters of the imaging setup can be tuned to achieve different contrast. For example, changing the distance between the sample and the detector (CL) in ADF mode can be used for the detection of TEs scattered at different angles (so-called low-angle, mid-angle, and high-angle ADF imaging).

Therefore, it was demonstrated that the current design can be used as a flexible platform for performing a wide range of SEM experiments. Different configurations and further modifications can be discussed. The splitting of the sensors into different channels will create a segmented detector that can be used in a large variety of SEM experiments. For example, a 4-quadrant BSE detector can be utilized for the differentiation between topographical and compositional contrasts using a combination of the signals from different segments [13]. The aforementioned configuration of the BSE detector also finds its application in the 3D reconstruction of the surface based on the shape-from-shading technique [14], [15]. Placing the photodiode on the path of the primary electron beam will allow using the detector for bright-field imaging. Coupled with the ADF detector, proposed configuration becomes a versatile platform for performing TSEM experiment.

## CRediT authorship contribution statement

**Evgenii Vlasov:** Conceptualization, Methodology, Writing – original draft. **Nikita Denisov:** Validation, Investigation, Data curation, Writing – original draft. **Johan Verbeeck:** Conceptualization, Supervision, Project administration.

## Declaration of Competing Interest

The authors declare that they have no known competing financial interests or personal relationships that could have appeared to influence the work reported in this paper.
